# Moisture retention of glycerin solutions with various concentrations: a comparative study

**DOI:** 10.1038/s41598-022-13452-2

**Published:** 2022-06-17

**Authors:** H. J. Chen, P. Y. Lee, C. Y. Chen, S. L. Huang, B. W. Huang, F. J. Dai, C. F. Chau, C. S. Chen, Y. S. Lin

**Affiliations:** 1 Department of Food Science and Biotechnology, National Chung Hsing University, No. 145, Xingda Rd., South Dist., Taichung City, 402204 Taiwan, ROC; 2 Healthmate Co., Ltd., No. 14, Pinghe 1st St., Changhua City, 500016 Taiwan, ROC; 3 Department of Optoelectronics and Materials Technology, National Taiwan Ocean University, No. 2, Beining Rd., Zhongzheng Dist., Keelung City, 202301 Taiwan, ROC; 4 Ph.D. Program in Materials and Chemical Engineering, National United University, No. 2, Lienda Rd., Miaoli City, 360302 Taiwan, ROC; 5 Department of Chemical Engineering, National United University, No. 2, Lienda Rd., Miaoli City, 360302 Taiwan, ROC; 6 Institute of Food Safety and Health Risk Assessment, National Yang Ming Chiao Tung University, No. 155, Sec. 2, Linong St. Beitou Dist., Taipei City, 112304 Taiwan, ROC

**Keywords:** Chemistry, Engineering

## Abstract

Various methods of evaluating a humectant’s moisture retention have unique mechanisms. Hence, for designing advanced or efficient ingredients of cosmetic products, a clear understanding of differences among methods is required. The aim of this study was to analyze the moisture-retention capacity of glycerin, a common ingredient in cosmetic products. Specifically, this study applied gravimetric analysis, transepidermal water loss (TEWL) analysis, and differential scanning calorimetry (DSC) to examine the evaporation of glycerin solutions of different concentrations. The results revealed that the moisture-retention capacity of glycerin increased with the glycerin concentration from 0 to 60 wt%, and glycerin at concentration of 60–70 wt% did not exhibit weight change during the evaporation process. When the glycerin concentration exceeded 70 wt%, moisture sorption occurred in the glycerin solution. Furthermore, the results revealed a deviation between the evaporation rates measured using gravimetric analysis and those measured using TEWL analysis. However, normalizing the results of these analyses yielded the relative evaporation rates to water, which were consistent between these two analyses. DSC thermograms further confirmed the consistent results and identified two hydrated water microstructures (nonfreezable water and free water) in the glycerin solutions, which explained why the measured evaporation rate decreased with the glycerin concentration. These findings can be applied to prove the moisture-retention capacity of a humectant in cosmetic products by different measuring methods.

## Introduction

The moisture-retention capacity of ingredients is crucial in cosmetics^1^. An effective moisture-retaining agent in cosmetic products can be beneficial against skin aging^2,3^. A humectant is a hygroscopic substance that can maintain skin moisture and hydration^3,4^. Loss of skin hydration engenders skin dryness, wrinkling, sagging, and laxity. Accordingly, several studies have sought humectants that exhibit high efficacy in retaining moisture on the human stratum corneum^5^.

A humectant’s moisture-retention capacity can be measured through various methods such as gravimetric analysis, transepidermal water loss (TEWL) analysis, differential scanning calorimetry (DSC), thermogravimetric analysis, dilatometry, infrared spectroscopy, and nuclear magnetic resonance spectroscopy–based relaxation time analysis^6–9^. Among these methods, gravimetric analysis can be easily applied to measure the weight change of an analyte in a material through evaporation within a specific period; a low level of weight loss indicates high moisture retention. However, because of the detection limit of balance machines used for gravimetric analysis, considerable time is required for accumulating detectable weight changes in order to measure a solution’s evaporation rate, which is an indicator of the solution’s moisture-retention^10^. Therefore, in gravimetric analysis, obtaining accurate evaporation rates is a time-consuming process^11^.

In general, TEWL refers to the amount of water vapor that permeates a certain area of membrane per unit of time and can be measured using a probe. A TEWL probe is an open-chamber system that applies two pairs of temperature and moisture sensors on a cylinder to determine water loss (in grams per hour per square meter) through evaporation^12^. The measuring principle of a TEWL probe is based on Fick’s law of diffusion, which relates to the mass transfer rate of water per unit area within a specific period. Compared with water loss measurement methods that involve weighing an analyte, a TEWL probe can afford a more stable measurement of water loss in a few minutes^13^.

**Figure 1:**
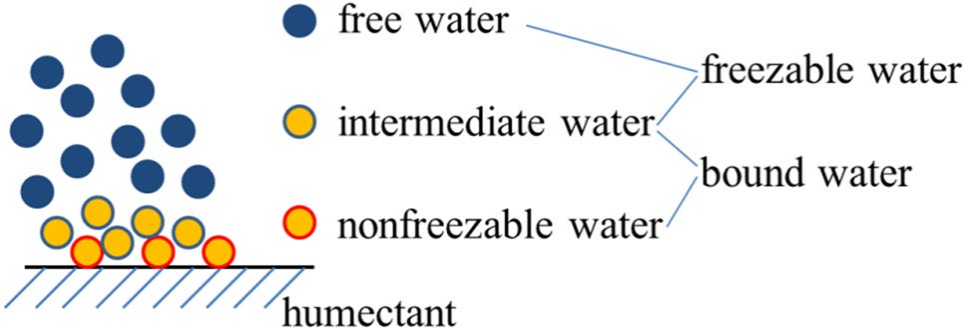
Three hydrated water types in a humectant.

DSC is a powerful tool for exploring the microstructure and thermal behavior of a liquid sample^14^; it can also be applied for evaluating the moisture retention of a humectant^15^. According to the freezing temperature criterion, the microstructure of water in a humectant can be categorized into three types: nonfreezable water, intermediate water, and free water^8,16–19^, as shown in Fig. 1 for three hydrated water types. Nonfreezable water and intermediate water can easily bind to a humectant through hydrogen bonding and are thus called bound water. Intermediate water and free water can exhibit phase transitions and are thus called freezable water^20^. Nonfreezable water tightly binds to the hydrophilic sites of a humectant and has low mobility because of the strong water–humectant interactions. Specifically, nonfreezable water involves very weak free water–water interactions. Intermediate water is oriented around nonfreezable water and the humectant as a hydration shell, forming cage-like structures through which the maximum number of hydrogen bonds is achieved in the available space^21^. The molecular interactions of intermediate water involve both water–humectant and water–water interactions. Molecular interactions of free water mainly involve water–water interactions.

Various methods of evaluating a humectant’s moisture retention have unique mechanisms. Hence, for designing advanced or efficient ingredients of cosmetic products, a clearer understanding of the differences among such methods is required. Accordingly, this study used glycerin—a common humectant—as a model to examine moisture retention; specifically, the study examined the moisture-retention capacity of glycerin solutions of different concentrations by using three convenient methods, namely; gravimetric analysis, TEWL assessment and DSC, for comparison.

## Materials and methods

Glycerin (First Cosmetics Manufacture Co., Ltd., Taiwan) and deionized water were used in this study. Glycerin solutions of different concentrations (wt%) were prepared by diluting glycerin with various amounts of deionized water; these solutions were then subjected to evaporation experiments. Each evaporation experiment was conducted by placing 3 mL glycerin solution in a vial with an internal diameter of 9 mm. These experiments were conducted in a closed system at 30 °C and 70% relative humidity.

The weight change of the glycerin solutions during evaporation was automatically monitored using a precise five-digit electronic balance machine (AS 60/220.R2, Radwag Wagi Elektroniczne, Poland) for 35 h. Additionally, a well-known TEWL probe (Courage + Khazaka Electronic, Köln, Germany) was used to detect the evaporation rate of the glycerin solutions at the beginning of the evaporation process according to the international guidelines. A single measurement was collected every 2 s until the standard deviation was below 0.1 g/hr/m^2^.

DSC experiments were performed using a differential scanning calorimeter (Q10, TA Instruments, New Castle, USA) with a Thermo Model FC100AX0TA refrigerated cooling system and Thermal Advantage Universal Analysis software. A 5-mg sample was weighed and sealed in the aluminum pan of the calorimeter. The sample pan along with a reference pan was then placed in the DSC instrument, cooled from 40 to − 50 °C, and heated up again to 40 °C at a rate of 1 °C/min to avoid the response time lag caused by a faster heating rate. The temperature and enthalpy peak associated with the phase transition during the heating process were analyzed. The enthalpy in unit of J/g was calculated by integration of enthalpy peak and normalization of water weight in the glycerin solution^6^. The experiments were repeated at least three times to ensure the reproducibility of the DSC results.

## Results and discussion

### Gravimetric analysis

**Figure 2:**
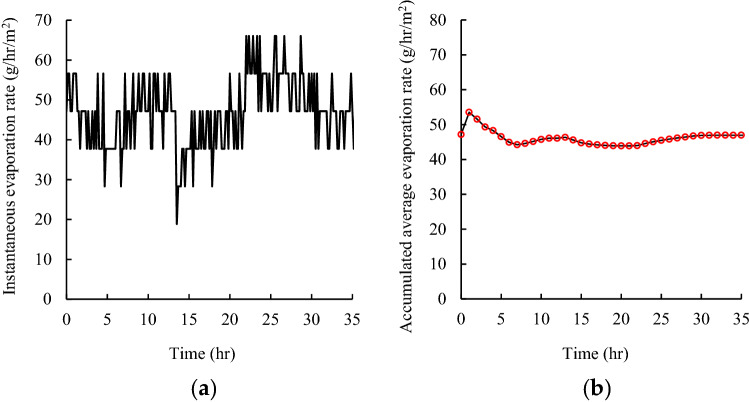
Gravimetric analysis of water evaporation rate with time: (**a**) instantaneous evaporation rate and (**b**) accumulative average evaporation rate.

Figure 2a illustrates the fluctuation of the instantaneous evaporation rate of a water solution with time; the rate was measured using an electronic balance. The weight of the water solution was measured automatically every minute during the evaporation process to calculate the instantaneous evaporation rate. The instantaneous evaporation rate fluctuated considerably because of a limited change in the weight of the water solution during the evaporation process and detection limitation of the balance. Figure 2b displays the accumulative average evaporation rate defined as the overall evaporation rate from start to a certain time, presented in Fig. 2a. As indicated in this figure, the accumulative average evaporation rate also fluctuated considerably during the early phases of the evaporation process due to the small weight change; however, the fluctuation decreased gradually with evaporation time because of the relatively large accumulated weight change. A stable accumulative average evaporation rate may be obtained after more than 5 h. Therefore, the gravimetric analysis was determined to consume considerable time before yielding a stable evaporation rate.

### Evaporation rates measured using gravimetric and TEWL analyses

**Figure 3:**
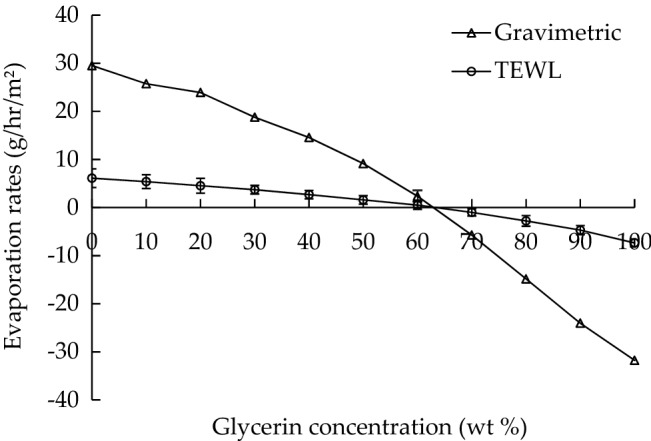
Evaporation rate of glycerin solutions of various concentrations measured by using gravimetric and TEWL analyses.

The evaporation rate of a humectant can be an indicator of the moisture-retention capacity of the humectant. Figure 3 presents evaporation rates measured through gravimetric and TEWL analyses for glycerin solutions of different concentrations (wt%). The evaporation rate of 10 wt% glycerin measured through TEWL analysis was determined to be consistent with that revealed by an in vivo report on 20 healthy volunteers^22^. The results of the two analyses indicated that the evaporation rate decreased with the glycerin concentration, demonstrating that a concentrated glycerin solution has a high moisture-retention capacity. No obvious evaporation rate could be measured when the glycerin concentration was at 60–70 wt%. This phenomenon can be attributed to the equilibrium between glycerin evaporation and moisture sorption. A glycerin molecule has three hydroxyl groups and is hygroscopic. When the glycerin concentration exceeded 70 wt%, a considerable amount of moisture sorption occurred, resulting in an increase in the weight of the glycerin solution and a negative evaporation rate.

This study revealed a deviation between the evaporation rates measured using gravimetric analysis and those measured using TEWL analysis. The rates measured using gravimetric analysis were higher than those measured using TEWL analysis. This deviation can be attributed to the different mechanisms of these two analyses. In gravimetric analysis, the direct evaporation rate of a solution is measured in terms of weight loss (in grams per hour per square meter) during the evaporation process. By contrast, in TEWL analysis, evaporation rate is evaluated as the rate of water vapor diffusion through a TEWL probe, as determined through the calculation of vapor density gradient using Fick’s law of diffusion.

**Figure 4:**
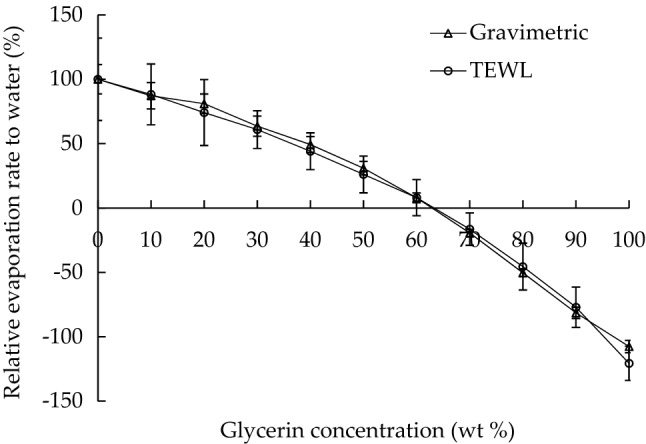
RERWs of glycerin solutions of various concentrations measured by using gravimetric and TEWL analyses.

To ensure a fair comparison between the analyses, this study normalized their results. The relative evaporation rate to water (RERW) was defined as the ratio of the water evaporation rate of glycerin solution to the water evaporation rate of pure water. Figure 4 displays the RERW measured using gravimetric and TEWL analyses. The rates derived from the two analyses were consistent, verifying the accuracy of this evaporation experiment. According to the definition of RERW, moisture sorption started when the RERW was less than 0%, where no water loss occurred. Therefore, as revealed in Fig. 4, when the RERW was 0%, the glycerin concentration was approximately 60–70 wt%. Glycerin concentrations that were lower than 60 wt% were associated with positive and less than 100% RERWs, indicating that glycerin at this concentration can achieve moisture retention and reduced evaporation. However, when the glycerin concentration was higher than 70 wt%, the RERW became negative, demonstrating that glycerin at this concentration can gain water. This finding agrees with the reports of Fluhr et al.^23^ and Kiran et al.^24^ that glycerin is an excellent humectant and hygroscopic agent. Humectancy or hygroscopicity is the tendency of a substance to absorb moisture from the surrounding atmosphere. Pure glycerin absorbs its own weight in water over 3 days^23^.

### DSC analysis

**Figure 5:**
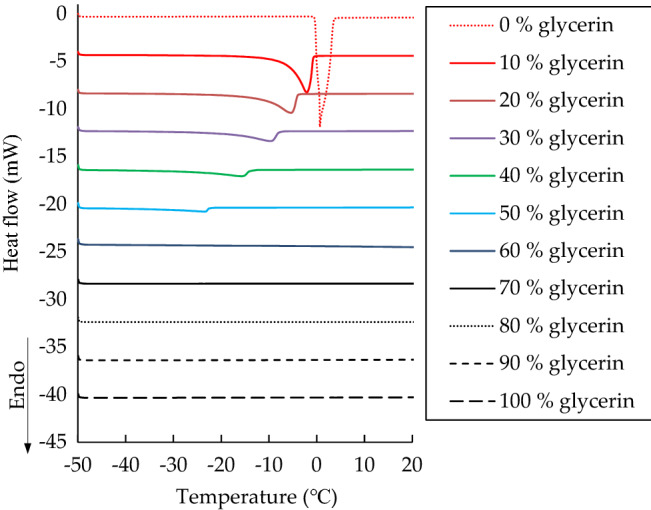
Heating curves of DSC thermograms at a 1 °C/min scanning rate for glycerin solutions of various concentrations.

DSC analysis was conducted to investigate the microstructure of water in the glycerin solutions. Figure 5 displays DSC thermograms of the glycerin solutions of different concentrations. The melting curves varied considerably with the glycerin concentration, with an obvious peak appearing at a glycerin concentration of 0 wt% and no signal appearing after a glycerin concentration of 70 wt% indicating existence of nonfreezable water. These peaks were ascribed to the melting of frozen water including bulk water and free water^18^. Different types of frozen water have different transition temperatures and peak shapes. The transition temperature of intermediate water is lower than that of free water^19^. Nevertheless, no melting peak was observed for intermediate water in this study. This result corresponded to a previous study reporting that poly(2-methoxyethylacrylate) analogous polymers had just two hydrated water types, nonfreezable water and free water^18^.

**Figure 6:**
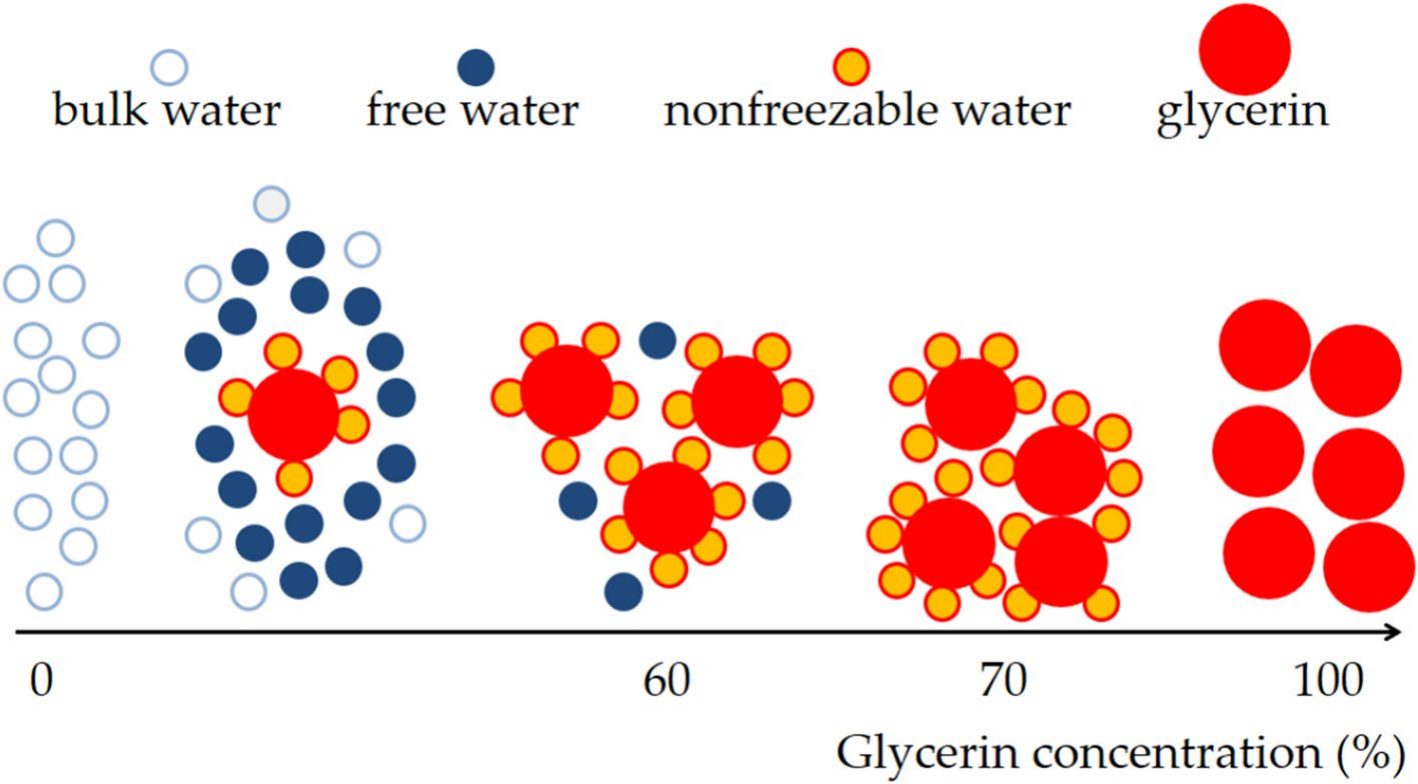
A schematic diagram to illustrate the microstructure type of the water in the glycerin solutions with various concentrations.

Table 1 presents a summary of the peaks observed for the glycerin solutions of different concentrations. The melting enthalpy observed for 0 wt% glycerin was noted to be consistent with the value obtained for pure water in a previous study^25^, indicating that the DSC method and conditions considered in the present study could be applicable to other study settings. The results also revealed that the peak temperature decreased with the glycerin concentration and that only 0 wt% glycerin was associated with a positive peak temperature. The positive melting peak indicates that the microstructure type of the water in 0 wt% glycerin was bulk water^18^. However, the melting peaks associated with 10–60 wt% glycerin were lower than 0 °C, signifying that the microstructure type of the water in the material also included free water except bulk water. When the glycerin concentration exceeded 70 wt%, no melting peak was observed, revealing that the microstructure of the water was nonfreezable water. Figure 6 illustrates the microstructure type of the water in the glycerin solutions at different concentrations.

**Table 1:** DSC thermogram analysis of 0 to 100 wt% glycerin solutions.

Glycerin concentration (wt%)	Peak temperature (°C)	Enthalpy (J/g)
0	0.64	334.3
10	− 2.28	213.8
20	− 5.48	167.0
30	− 9.80	112.1
40	− 15.77	67.7
50	− 23.50	50.8
60	− 32.45	8.5
70	–	–
80	–	–
90	–	–
100	–	–

The melting enthalpy peak decreased with the glycerin concentration, and no melting enthalpy was observed when the glycerin concentration exceeded 70 wt% (Table 1). This finding was consistent with the results of the evaporation experiments conducted using gravimetric analysis and the TEWL probe. The melting enthalpy resulted from frozen water (bulk water and free water), which can evaporate. The melting enthalpy increases with the amount of frozen water evaporating. This thus explains why the evaporation rate of the glycerin solutions decreased with the glycerin concentration. For concentrated glycerin solutions, the microstructure of the water tended to be nonfreezable water without evaporation.

**Figure 7:**
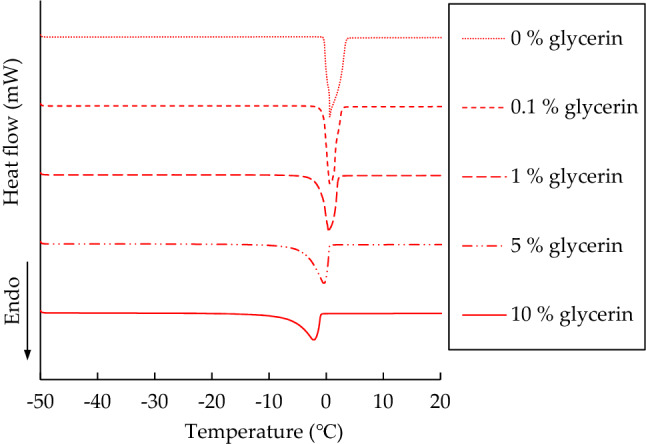
Heating curves of DSC thermograms at a 1 °C/min scanning rate for glycerin solutions with the concentrations of 0–10 wt%.

To more clearly demonstrate the microstructures of water, the DSC thermograms for glycerin solutions with concentrations of < 10 wt% are displayed in Fig. 7 for comparison. The curves for 0.1, 1, and 5 wt% glycerin were between those for 0 and 10 wt% glycerin. The melting peaks associated with 0.1, 1, and 5 wt% glycerin shifted left from 0 wt% glycerin toward lower temperature regions; additionally, the melting temperatures ranged from both above and below 0 °C. This phenomenon signifies that free water was formed when glycerin molecules were added to the bulk water solution^18^. When the glycerin concentration reached 10 wt%, the melting peak was in the negative temperature region because of the large amount of free water. Furthermore, as revealed in Table 2, in addition to the peak temperature, the melting enthalpy decreased with the glycerin concentration.

**Table 2:** DSC thermogram analysis of glycerin solutions with low concentrations.

Glycerin concentration (wt%)	Peak temperature (°C)	Enthalpy (J/g)
0	0.64	334.3
0.1	0.48	295.3
1	0.42	269.4
5	− 0.68	259.8
10	− 2.28	213.8

## Conclusions

This study compared three methods used to evaluate the moisture-retention capacity of glycerin solutions of different concentrations. The results indicate that the moisture-retention capacity of glycerin increases with the glycerin concentration. Although a deviation was observed between the results of gravimetric analysis and TEWL analysis, normalizing the results of these analyses revealed reasonably high consistency levels between them. In addition to confirming the consistency between the gravimetric and TEWL analyses results, this study generated DSC thermograms to further identify two hydrated water forms in the glycerin solutions, which explained the measured evaporation rates of the glycerin solutions. These findings can be applied to prove the moisture-retention capacity of a humectant in cosmetic products by different measuring methods.

## Data Availability

All data generated or analysed during this study are included in this published article.
